# Genome-wide identification of loci associated with growth in rainbow trout

**DOI:** 10.1186/s12864-020-6617-x

**Published:** 2020-03-05

**Authors:** Ali Ali, Rafet Al-Tobasei, Daniela Lourenco, Tim Leeds, Brett Kenney, Mohamed Salem

**Affiliations:** 1 Department of Animal and Avian Sciences, University of Maryland, College Park, MD 20742 USA; 2 Computational Science Program, Middle Tennessee State University, Murfreesboro, TN 37132 USA; 3 Department of Animal and Dairy Science, University of Georgia, Athens, GA 30602 USA; 4 United States Department of Agriculture Kearneysville, National Center for Cool and Cold Water Aquaculture, Agricultural Research Service, Kearneysville, WV USA; 5 Division of Animal and Nutritional Sciences, West Virginia University, Morgantown, WV 26506 USA

**Keywords:** Body weight, Fish, Genomic selection, QTL, GWAS, WssGBLUP

## Abstract

**Background:**

Growth is a major economic production trait in aquaculture. Improvements in growth performance will reduce time and cost for fish to reach market size. However, genes underlying growth have not been fully explored in rainbow trout.

**Results:**

A previously developed 50 K gene-transcribed SNP chip, containing ~ 21 K SNPs showing allelic imbalances potentially associated with important aquaculture production traits including body weight, muscle yield, was used for genotyping a total of 789 fish with available phenotypic data for bodyweight gain. Genotyped fish were obtained from two consecutive generations produced in the NCCCWA growth-selection breeding program. Weighted single-step GBLUP (WssGBLUP) was used to perform a genome-wide association (GWA) analysis to identify quantitative trait loci (QTL) associated with bodyweight gain. Using genomic sliding windows of 50 adjacent SNPs, 247 SNPs associated with bodyweight gain were identified. SNP-harboring genes were involved in cell growth, cell proliferation, cell cycle, lipid metabolism, proteolytic activities, chromatin modification, and developmental processes. Chromosome 14 harbored the highest number of SNPs (*n* = 50). An SNP window explaining the highest additive genetic variance for bodyweight gain (~ 6.4%) included a nonsynonymous SNP in a gene encoding inositol polyphosphate 5-phosphatase OCRL-1. Additionally, based on a single-marker GWA analysis, 33 SNPs were identified in association with bodyweight gain. The highest SNP explaining variation in bodyweight gain was identified in a gene coding for thrombospondin-1 (THBS1) (*R*^2^ = 0.09).

**Conclusion:**

The majority of SNP-harboring genes, including OCRL-1 and THBS1, were involved in developmental processes. Our results suggest that development-related genes are important determinants for growth and could be prioritized and used for genomic selection in breeding programs.

## Background

Aquaculture is a growing agribusiness that enhances food security and increases economic opportunities worldwide [1]. A key challenge for this industry is to sustain the increasing consumer demand for seafood [2]. Salmonid species have been extensively studied as cultured fish species due to their economic and nutritional value [3]. Growth performance, particularly the efficiency of converting feed to bodyweight gain, is one of the most economically important traits [3]. Growth is a complex trait controlled by environmental and genetic factors. Despite the multi-environmental factors that may affect growth, quantitative genetics studies revealed moderate to high levels of growth rate heritability [4, 5]. Thus, artificial selection for growth is plausible, allowing potential improvement through selective breeding programs [5].

Selective breeding improves heritable traits, taking advantage of existing genetic variation between individuals/families. Previous studies showed that selective breeding programs can improve animals’ bodyweights, thereby contributing to increased aquaculture production [6, 7]. Selection on harvest weight can improve growth rate [8] and flesh color, and reduce production cost [9]. Successful genetic programs depend on the establishment of a base population with natural genetic variation, which helps to achieve a long-term response to selection. A family-based selection line for growth was established in 2002 at the USDA National Center for Cool and Cold Water Aquaculture (NCCCWA). Five generations of selection yielded a 10% gain in bodyweight per generation [10] at harvest. More efforts are required to understand the genetic basis of bodyweight gain for genetically improved strains to achieve fast/efficient production [2].

QTL mapping has been extensively applied in plants and farmed animals to determine the genetic architecture of the complex traits. Several QTL mapping studies were performed to assess the genetic basis of growth in Atlantic salmon, Coho salmon, and rainbow trout [3]. For instance, a significant QTL for body weight was co-localized with another moderate-effect QTL for maturation timing in the linkage group RT-27 in rainbow trout [11–13]. In addition, QTL for body weight and condition factor were co-localized on linkage group RT-9 and RT-27 [4]. However, classical QTL mapping has some limitations. Linkage analysis is time-consuming and depends on the segregation of alleles within a family, limiting the power to detect associations between markers and phenotypes of interest [5]. In addition, the identified QTL encompasses several megabases that contain hundreds, if not thousands, of genes, making it challenging to identify the causal gene in a QTL [14].

Genomic resources have been developed for rainbow trout, including the release of the first genome assembly draft [15] and a newly assembled genome (GenBank assembly, NCBI accession GCA_002163495, RefSeq assembly accession GCF_002163495). New sequencing technologies have identified SNPs that are widely distributed throughout the genome; this SNP distribution enabled the construction of high-density genetic maps [16, 17]. About 90% of the genetic variation comes from SNPs that are highly adaptable to large-scale genotyping and, therefore, most suitable for GWA studies [8]. The rainbow trout genome was successfully used for calling variants [18], and these variants have been used to build a 50 K transcribed gene SNP chip suitable for association mapping [19]. GWA studies have been employed to test the association between SNP markers spread throughout the genome and complex quantitative traits of interest [20]. Owing to the drastic reduction in cost and time required for genotyping a large number of markers, GWA studies are replacing QTL linkage mapping [21]. SNP markers in linkage disequilibrium (LD) with QTL associated with the trait of interest could be identified from GWA analyses and prioritized in selective breeding programs [20]. Many GWA studies conducted on livestock species led to the identification of genes and mutations associated with economic traits [20]. Recently, a few GWA studies have been implemented in aquaculture species [20], including rainbow trout. These studies aimed to identify markers associated with bodyweight [22], fillet quality [19, 22], and disease resistance [23]. Growth traits are controlled by small-effect variants in the farmed Atlantic salmon [24]. In addition, a recent GWA study using a 57 K SNP array identified QTL explaining a small proportion of additive genetic variance for body weight in rainbow trout. A single window on chromosome 5 was responsible for 1.4 and 1.0% of the additive genetic variance in body weight at 10 and 13 months post-hatching, respectively [22].

In this study, we used a 50 K transcribed gene SNP chip, recently developed in our laboratory, to perform GWA analyses [19]. The chip has 21 K SNPs of potential associations with muscle growth, fillet quality, and disease resistance traits. In order to randomize SNP distribution in this chip, 29 K additional SNPs were added to the chip following a strategy of 2 SNPs per each SNP-harboring gene. The SNP chip has been successfully used to identify QTL associated with muscle yield [19], and fillet firmness and protein content [25] in rainbow trout. The objective of this study was to use the 50 K SNP array to identify large-effect QTL associated with the growth rate that could be applied in genomic selection.

## Results and discussion

**Fig. 1:**
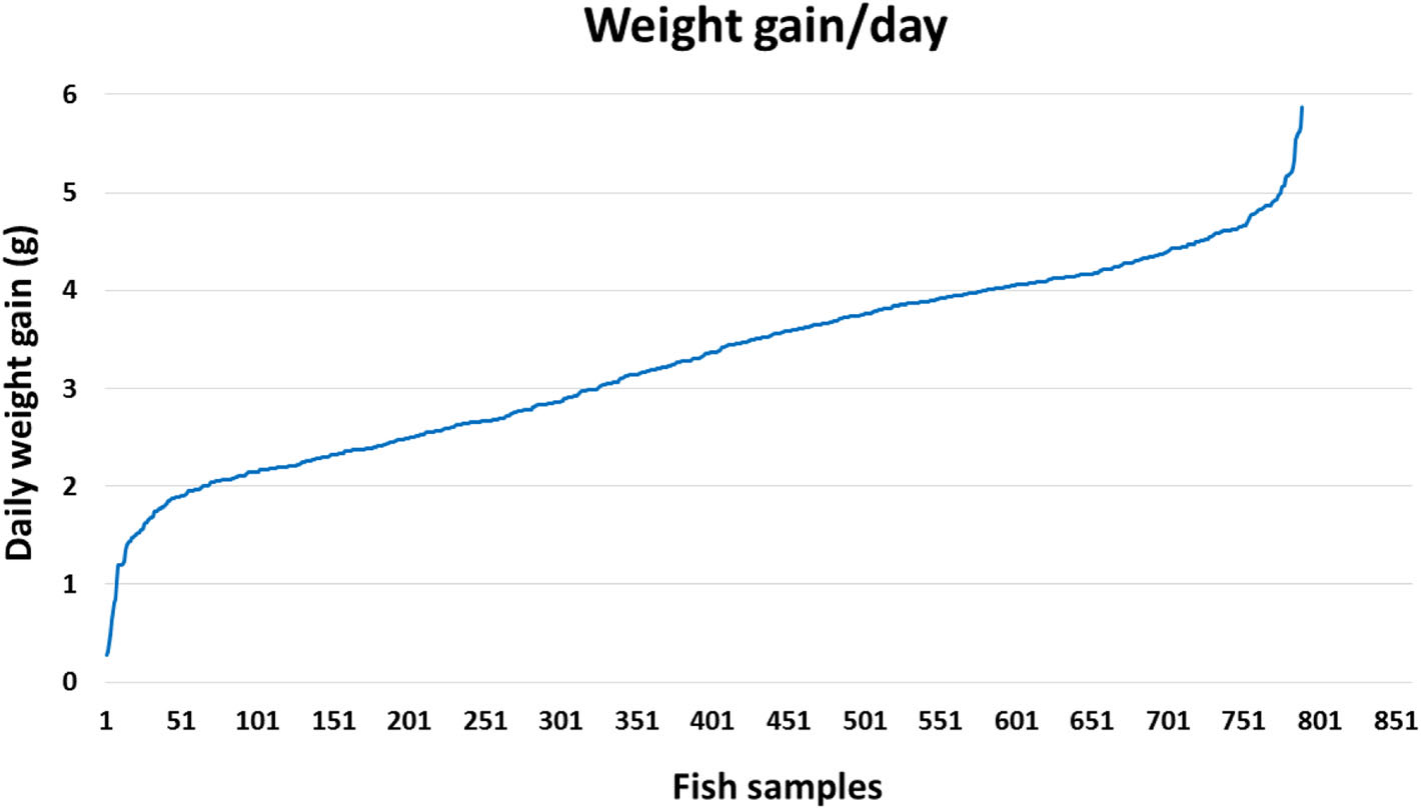
Variations in bodyweight gain among fish samples used in GWA analysis

Growth performance defines fish production, and therefore, it affects aquaculture industry profitability. Progress in growth-related traits could lead to reductions in time and cost to market size [26]. Traditional selection, based on the phenotype, has been applied to select for growth traits resulting in approximately 10% gain in body weight per generation [10]. The economic significance of growth to aquaculture encouraged several studies aimed at understanding the genetic basis/mechanisms underlying the phenotype [26]. Genomic approaches have the potential to expedite genetic gains compared to traditional selection. SNPs account for 90% of sequence variants in humans [27]; therefore, SNPs are most suitable for genetic evaluation of breeding candidates in selection programs. The fish population used for the current GWA analysis had an average bodyweight gain per day of 3.27 ± 0.96 (g). Variations in bodyweight gain among 789 fish used for the current GWA analysis are shown in Fig. 1. The estimated heritability for bodyweight gain in rainbow trout was 0.30 ± 0.05. In this study, a 50 K SNP chip was used to identify genomic regions associated with bodyweight gain, based on 50 SNP sliding windows and single-marker association analysis. It is worth mentioning that a total of 90 fish from YC2010 were used in our previous study [18] to identify putative SNPs associated with muscle growth and quality traits (WBW, muscle yield, fat content, shear force, and whiteness index). The putative SNPs showing allelic imbalance (7.9 K SNPs) with the five growth and quality traits were included in the SNP chip [19]. To make sure those fish do not interfere with the GWAS results, those 90 fish were excluded from the analysis in this study.

### Identifying QTL associated with bodyweight gain using WssGBLUP

**Fig. 2:**
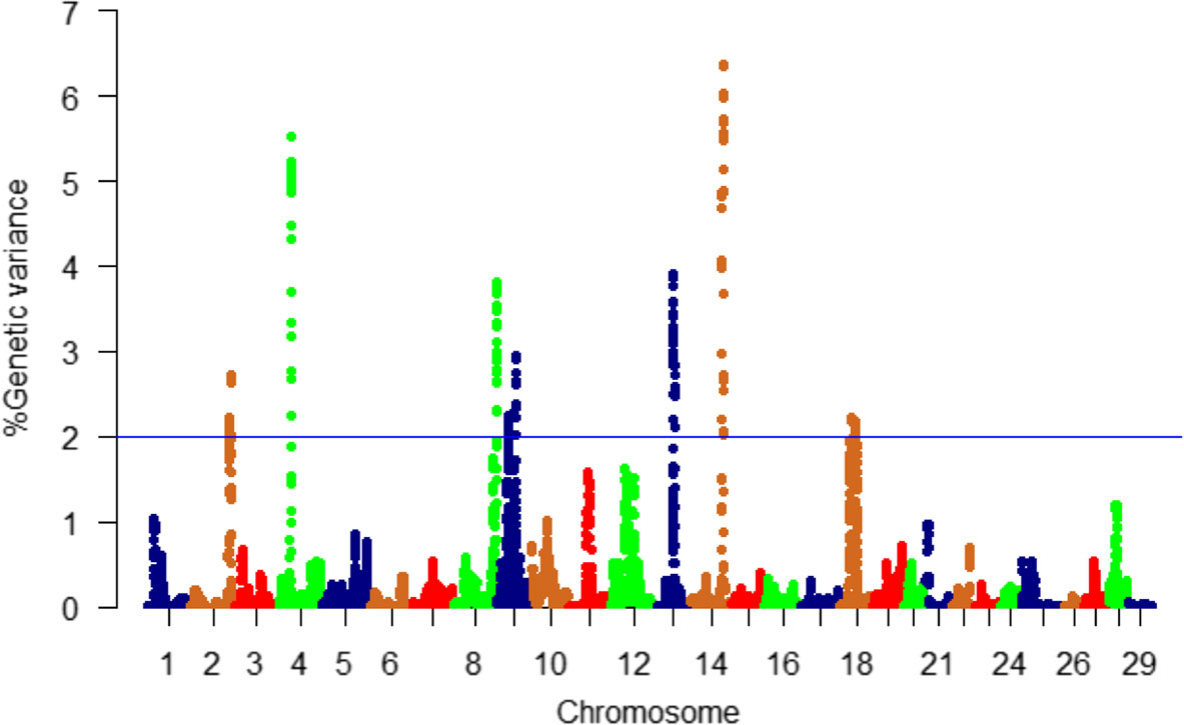
Manhattan plot displaying the association between genomic sliding windows of 50 SNPs and bodyweight gain. Chromosome 14 showed the highest peaks with genomic loci explaining up to 6.37% of the additive genetic variance. The blue line represents 2% of additive genetic variance explained by SNPs

WssGBLUP-based GWA analysis identified a total of 247 SNPs associated with additive genetic variance in bodyweight gain. These SNPs exist in 107 protein-coding genes, 6 lncRNAs, and 36 intergenic regions. SNPs were identified in windows explaining at least 2% (arbitrary value) of the additive genetic variance for bodyweight gain (Table S1). The genomic regions that harbor SNPs were clustered on 7 chromosomes (2, 4, 8, 9, 13, 14, and 18) (Fig. 2). Chromosome 14 had the most significant peaks associated with bodyweight gain (up to 6.37%) and the highest number of SNPs (*n* = 50) in windows explaining additive genetic variance for the studied trait (Table S1, Fig. 2). Many of the SNPs (*n* = 100) were located within the 3’UTR of their genes suggesting a role of these SNPs in microRNA, post-transcriptional regulation of gene expression. All QTLs associated with bodyweight gain are listed in Table (S1). To gain understandings into the biological significance of the identified QTL, we annotated SNP-harboring genes and followed this annotation by gene enrichment analysis. Functional annotation analysis showed that SNP-harboring genes were involved in cell growth, cell cycle, cell proliferation, lipid metabolism, proteolytic activities, developmental processes, and chromatin modification. Enriched terms included lysosomal proteins/enzymes and fatty acid biosynthesis (Table S2).

### SNPs in genes regulating cell growth, cell cycle and cell proliferation

Coordinated hypertrophy and hyperplasia are essential for growing organisms [28]. Five chromosomes (2, 4, 9, 13, and 14) had SNPs regulating cell growth, cell cycle, and cell proliferation (Table 1). Chromosome 2 had 14 SNPs in 6 genes coding for caveolin-1 (CAV-1), testin (TES), eukaryotic translation initiation factor 4 gamma 2 (EIF4G2), sodium-dependent neutral amino acid transporter B (0) AT2 (SLC6A15), kinesin-like protein KIF21A (KIF21A), and G1/S-specific cyclin-D1 (CCND1). Six SNPs spanning ~ 1.8 Kb were identified in CAV-1. The latter has a role in inhibiting the activity of TGF-β, probably by enfolding TGF-β receptors in membrane invaginations [29]. Knockdown of CAV-1 had a tumor-suppressing effect by inhibiting cell proliferation [30], arresting cells in the G0/G1 phase, and inhibiting the expression of cell cycle-related proteins such as cyclin D1 [30]. Two SNPs were identified in each of TES and EIF4G2. TES negatively regulates cell proliferation and inhibits tumor cell growth [31, 32], whereas eIF4G2 positively regulates cell growth and proliferation, prevents autophagy, and releases cells from nutrient-sensing control by mTOR [33]. Each of SLC6A15 and KIF21A had a single SNP. Depletion of SLC6A15 attenuates leucine’s effects in reducing weight gain associated with a high-fat diet [34]. KIF21A has been identified in association with growth in pigs [35]. We identified 2 SNPs in the CCND1 gene. This cyclin is expressed during the G1 phase to signal initiation of DNA synthesis; it is suppressed during the S phase to allow DNA synthesis [36]. Cancer cell proliferation [37] and the growth of multifocal dysplastic lesions [38] were regulated through CCND1.

**Table 1:**
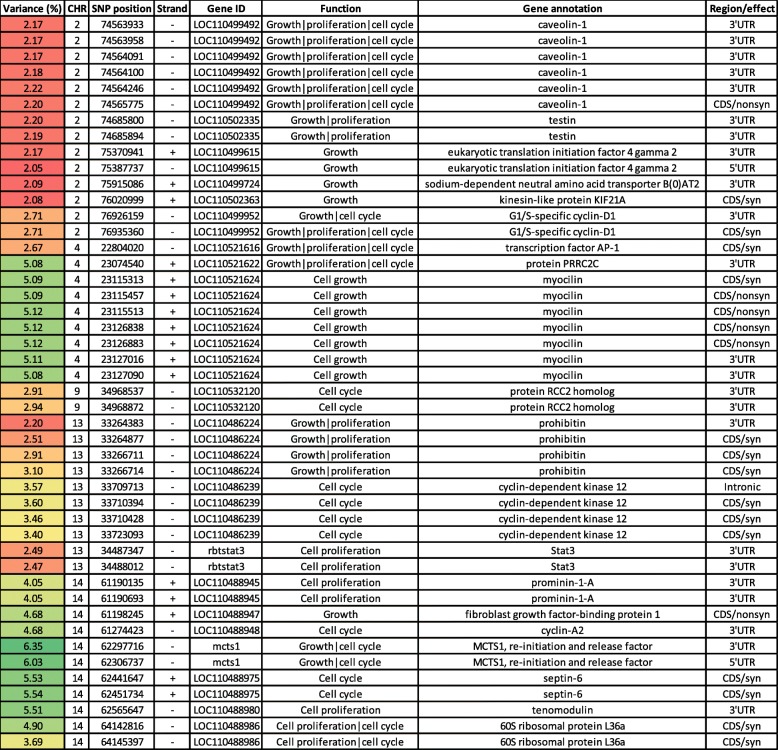
Genomic sliding windows of 50 SNPs explaining at least 2% of the additive genetic variance for bodyweight gain by affecting growth, cell cycle, and cell proliferation

A color gradient on the left indicates differences in additive genetic variance explained by windows containing the representative SNP marker (green is the highest and red is the lowest). SNPs are sorted according to their chromosome positions

A total of 21 SNPs were identified on chromosomes 4, 9, and 13. Chromosome 4 had 9 SNPs in 3 genes coding for transcription factor AP-1 (AP-1), protein PRRC2C (PRRC2C), and myocilin (MYOC). Transcription factor AP-1 transduces growth signals to the nucleus, mediated by upregulation of positive cell cycle regulators [39], which enhance the expression of genes involved in growth [40]. Whereas PRRC2C regulates the cell cycle and cell proliferation, and it controls the growth of lung cancer cells in vitro [41]. MYOC had 4 nonsynonymous SNPs. Transgenic mice, with 15-fold over-expressed MYOC, exhibited skeletal muscle hypertrophy with an approximate 40% increase in muscle weight [42]. We identified 2 SNPs on chromosome 9 in the gene coding for protein RCC2 homolog. RCC2 is a crucial regulator of cell cycle progression during the interphase [43]. There were ten SNPs in 3 genes on chromosome 13. Four SNPs, spanning 2.3 Kb, were localized in a gene coding for prohibitin (PHB). This protein suppresses cell growth by controlling E2F transcriptional activity [44]. Four SNPs spanned a gene coding for cyclin-dependent kinase 12 (CDK12). Depletion of CDK12 revealed increased numbers of accumulated cells at the G2/M phase and supported a role for CDK12 in maintaining genomic stability [45]. STAT3 had two SNPs in the 3’UTR. Knockdown of STAT3 inhibits cell proliferation and leads to irreversible growth arrest [46].

Chromosome 14 had 11 SNPs in seven genes coding for prominin-1-A (PROM1A), fibroblast growth factor-binding protein 1 (FGFBP1), cyclin A2 (CCNA2), re-initiation and release factor (MCTS1), septin-6 (SEPT6), tenomodulin (TNMD), and 60S ribosomal protein L36a (RPL36A). PROM1A has a role in cell proliferation and differentiation [47]. FGFBP1 promotes fibroblast growth factor2 (FGF2) signaling during angiogenesis, tissue repair, and tumor growth [48]. A single SNP was identified in the CCNA2 gene. This gene has a crucial role in cell cycle by regulating the initiation and progression of DNA synthesis [49]. The untranslated regions of a gene coding for MCTS1 had two SNPs in windows explaining up to ~ 6.4% of the additive genetic variance for bodyweight gain. Overexpression of MCTS1 promotes lymphoid tumor development leading to increased growth rates and protection against apoptosis [50]. In addition, MCTS1 is involved in cell cycle progression by decreasing the length of the G1 phase without a reciprocal increase in other phases [51]. Each of SEPT6 and RPL36A had 2 SNPs in windows associated with the additive genetic variance for bodyweight gain. Knockdown of SEPT6 leads to loss of cell polarity as a result of nuclear accumulation of the adaptor protein NCK, which arrests the cell cycle [52]. Over-expression of RPL36A leads to rapid cell cycling which enhances cell proliferation [53]. Of note, TNMD had an SNP in a window explaining 5.5% of the additive genetic variance. TNMD is essential for tenocyte proliferation and collagen fibril maturation [54]. Thirty-one genes involved in cell growth, cell cycling, and cell proliferation were differentially expressed (DE) in fish families (year class “YC” 2010), exhibiting divergent whole-body weight (WBW) phenotype. Of these genes, CAV was downregulated in families of high WBW relative to those of low WBW [55]. Our results indicate a role for increased biomass and cell numbers in explaining variations in body weight.

### SNPs in genes regulating lipid metabolism

Fatty acid synthesis is essential to meet the demand for phospholipids required for membrane expansion in growing cells [56]. We have identified 29 SNPs in 16 genes involved in lipid metabolism, explaining at least 2% of the additive genetic variance in bodyweight gain (Table 2). These SNPs spanned 5 chromosomes (4, 8, 13, 14, and 18). Chromosome 4 had 15 SNPs (56.6%) in 7 genes; peroxiredoxin 6 (PRDX6), phospholipid phosphatase 6 (PLPP6), vesicle-associated membrane protein 4 (VAMP4), phosphatidylinositol Glycan, Class C (PIGC), disabled homolog 1 (DAB1), AMPK subunit alpha-2 (PRKAA2), and phospholipid phosphatase 3 (PLPP3). Three SNPs were identified in the gene coding for PRDX6. The bifunctional enzyme, PRDX6, regulates phospholipid turnover as well as protects against oxidative injury [57]. A single 3’UTR SNP was identified in the VAMP4 gene. This gene encodes a protein implicated in the growth of lipid droplets in rainbow trout [58]. Also, the DAB1 had a 3’UTR SNP. DAB1 is associated with intramuscular fatty acid content in pigs [59]. PRKAA2 harbored 3 SNPs located within windows that were among those explaining the highest genetic variation in bodyweight gain. AMPK regulates lipid metabolism by inhibiting the activity of critical enzymes necessary for de novo biosynthesis of fatty acids and cholesterol [60]. PLPP3 had 5 SNPs in windows explaining ~ 5% of the additive genetic variance. This enzyme catalyzes the conversion of phosphatidic acid to diacylglycerol, which is vital to improving meat quality and lower body fat accumulation [61].

**Table 2:**
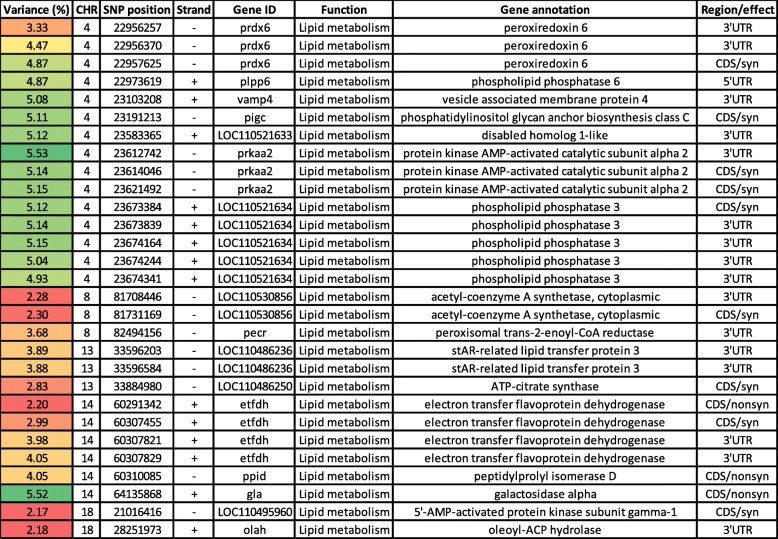
Genomic sliding windows of 50 SNPs explaining at least 2% of the additive genetic variance for bodyweight gain and involved in lipid metabolism.

A color gradient on the left indicates differences in additive genetic variance explained by windows containing the representative SNP marker (green is the highest and red is the lowest). SNPs are sorted according to their chromosome positions

In total, 14 SNPs were identified on chromosomes 8, 13, 14, and 18. Chromosome 8 had three SNPs in 2 genes encoding acetyl-coenzyme A synthetase (ACSS2) and peroxisomal trans-2-enoyl-CoA reductase (PECR). ACSS2 activates acetate that can be used for lipid synthesis [62]. In addition, the PECR contributes to chain elongation of fatty acids [63]. Chromosome 13 had 3 SNPs in genes coding for stAR-related lipid transfer protein 3 (STARD3) and ATP-citrate synthase (ACLY). STARD3 acts as a mediator of lipid metabolism and is required for the growth and survival of cancer cells [64]. A single coding SNP was identified in a gene coding for ACLY. This enzyme has a crucial role in de novo biosynthesis of lipids and promoting tumor growth [56]. Six SNPs were identified on chromosome 14 in genes coding for electron transfer flavoprotein dehydrogenase (ETFDH), peptidylprolyl isomerase D (PPID), and galactosidase alpha (GLA). Four polymorphic sites were identified in ETFDH. Mutations in ETFDH gene lead to a disorder of fatty acid, amino acid, and choline metabolism [65]. An SNP was identified in PPID gene that has gene ontology (GO) terms belonging to lipid particle organization. In addition, we identified two SNPs on chromosome 18 in genes encoding AMPK subunit gamma-1 (PRKAG1) and oleoyl-ACP hydrolase. The latter enzyme contributes to the release of free fatty acids from fatty acid synthase [66]. Moderate to high heritability for growth-related traits and fat content has been reported, implying the existence of additive genetic variance in the fish population [22, 67]. In fish from the YC 2010, one of the two generations of fish used in the study, fat content exhibited a moderate regression coefficient (*R*^2^) value of 0.50 with WBW [55]. Many genes (*n* = 31) involved in lipid metabolic processes, including AMPK, were DE in fish families (YC 2010), showing contrasting WBW [55]. These results suggest a substantial role for fat content in explaining variations in body weight.

### SNPs in genes regulating proteolytic activities

A total of 19 SNPs involved in proteolytic activities were identified in 12 genes (Table 3). Out of them, 9 SNPs were located on 4 genes involved in the KEGG lysosome pathway; lysosomal associated membrane protein 2 (LAMP2), V-type proton ATPase subunit H (ATP6V1H), galactosidase alpha (GLA), and neuraminidase 1 (NEU1). Five SNPs in LAMP2 have been identified in windows explaining the highest genetic variation (~ 6%) in this category. LAMP2 is essential during autophagy for the fusion of autophagosomes with lysosomes [68]. ATP6V1H is a vacuolar (H+)-ATPase, which is required to acidify the phagosome/lysosome for proper processing [69]. GLA and NEU1 are lysosomal acid hydrolases (glycosidases) required to breakdown glycoproteins [70]. NEU1 was associated with suppression of ovarian carcinoma [71]. In addition, 9 SNPs were identified in 4 genes engaged in the phagosome pathway. These genes are encoding ras-related protein Rab-5C (RAB5C), ATP6V1H, LAMP2, and integrin beta-3 (ITGB3). An SNP on chromosome 4 was located in a gene coding for OMA1 zinc metallopeptidase (OMIM). The OMIM is a protease essential for mitochondrial inner membrane proteostasis maintenance [72], and its deficiency leads to increased body weight and obesity [73]. Plectin had two SNPs. Mutation in plectin results in muscular dystrophy [74]. In addition, we identified 5 SNPs located on 4 genes exhibiting peptidase activity; trypsin-3, carboxypeptidase A1, carboxypeptidase B2 (CPB2), and high choriolytic enzyme 2. Forty-three genes have functions related to protein metabolic processes and were DE in fish families (YC 2010) showing substantial variation in WBW [55]. These results support a role for protein turnover in determining body weight.

**Table 3:**
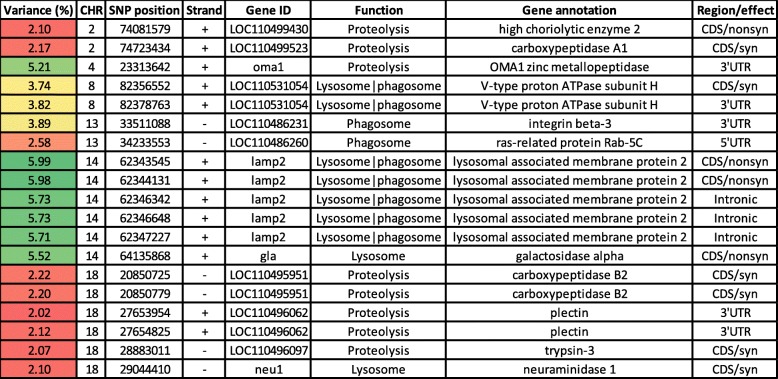
Genomic sliding windows of 50 SNPs explaining at least 2% of the additive genetic variance for bodyweight gain and involved in proteolytic activities

A color gradient on the left indicates differences in additive genetic variance explained by windows containing the representative SNP marker (green is the highest and red is the lowest). SNPs are sorted according to their chromosome positions

### SNPs in genes regulating developmental process and chromatin modification

Forty-five SNPs were identified in 21 genes involved in development and chromatin remodeling (Table 4 & Table S1). Chromosome 4 had 12 SNPs in five genes coding for phosphatidylinositol glycan anchor biosynthesis class C (PIGC), SUN domain-containing ossification factor (SUCO), transmembrane emp24 domain-containing protein 5 (TMED5), histone H2A deubiquitinase MYSM1 (MYSM1), and biogenesis of lysosome-related organelles complex-1 subunit 2 (BLOS2). PIGC encodes an endoplasmic reticulum membrane protein that has been linked to embryonic lethality [75]. Mutagenesis of SUCO leads to failure of osteoblast maturation, a decrease in the synthesis of type I collagen, and eventually catastrophic defects in skeletal development [76]. The gene encoding TMED5 has GO terms belonging to chromatin binding [77]. Knockdown of MYSM1, a histone H2A deubiquitinase, led to embryonic lethality and growth retardation [78]. BLOS2 harbored 6 SNPs in windows explaining up to 4.9% of the additive genetic variance. BLOS2 is a negative regulator of the Notch system, and lack of BLOS2 in mice was embryonic lethal and led to developmental defects [79]. We identified 6 SNPs on chromosomes 8 and 9. SNPs spanned three genes (2 SNPs/gene) encoding NADH dehydrogenase [ubiquinone] flavoprotein 2 (NDUFV2), ralA binding protein 1 (RALBP1), and short-chain dehydrogenase/reductase 3 (DHRS3). NDUFV2 is involved in nervous system development [77], whereas RALBP1 was involved in the regulation of actin dynamics during embryogenesis [80]. Knockdown of DHRS3 led to a phenotype with underdeveloped head structure and perturbed somitogenesis [81]. Chromosome 13 harbored the highest number of SNPs (*n* = 19) in this category. These SNPs were located in genes coding for methyltransferase-like protein 2-A (METTL2A), telethonin (TCAP), synaptonemal complex protein SC65 (SC65), peptidyl-prolyl cis-trans isomerase FKBP10 (FKBP10), 2′,3′-cyclic-nucleotide 3′-phosphodiesterase (CNP), and histone acetyltransferase KAT2A (KAT2A). METTL2A has GO terms belonging to methyltransferase activity [77]. Four SNPs were identified in TCAP. TCAP-null mice exhibit abnormal myofiber size variation and increased levels of TCAP binding protein, myostatin [82]. SC65 had two SNPs; whereas, FKBP10 had 4 SNPs. SC65 is expressed during skeletal development and acts as a regulator of bone mass homeostasis. Lack of SC65 leads to a progressive osteopenia [83]. Loss of function mutations in FKBP10 resulted in mice that were not able to survive birth, and embryos exhibited a growth delay and tissue fragility [84]. CNP had the highest number of SNPs on chromosome 13. This protein regulates blood supply to the developing embryo [85]. KAT2A encodes a protein that acts as a histone H3 succinyltransferase and exhibits a role in tumor cell proliferation and development [86]. KAT2A is involved in the regulation of developmental processes by mediating acetylation of TBX5 [87]. Six SNPs were identified on chromosome 14 in genes coding for Rap guanine nucleotide exchange factor 2 (RAPGEF2), glutathione S-transferase P (GSTP1), inositol polyphosphate 5-phosphatase OCRL-1 (OCRL), ETS-related transcription factor Elf-1 (ELF1), and mediator of RNA polymerase II transcription subunit 12 (MED12). OCRL was located in a window explaining the highest genetic variation in bodyweight gain (~ 6.4%), followed by ELF1 (~ 5.5%). Lacking both OCRL and its paralog (Inpp5b) led to the early lethality of mice embryos [88]. ELF1 has a role in maintaining cell polarity during development [89]. In addition, chromosome 18 had 2 SNPs in a gene encoding double-strand-break repair protein rad21 homolog (RAD21) (Table S1), which is involved in chromatin binding [77]. Sixty-three genes involved in development were DE in fish families (YC 2010) exhibiting divergent WBW phenotypes [55]. In agreement with a recent GWA study in rainbow trout [90], our results suggest a major role for genes involved in development in regulating genetic variation in bodyweight gain.

**Table 4:**
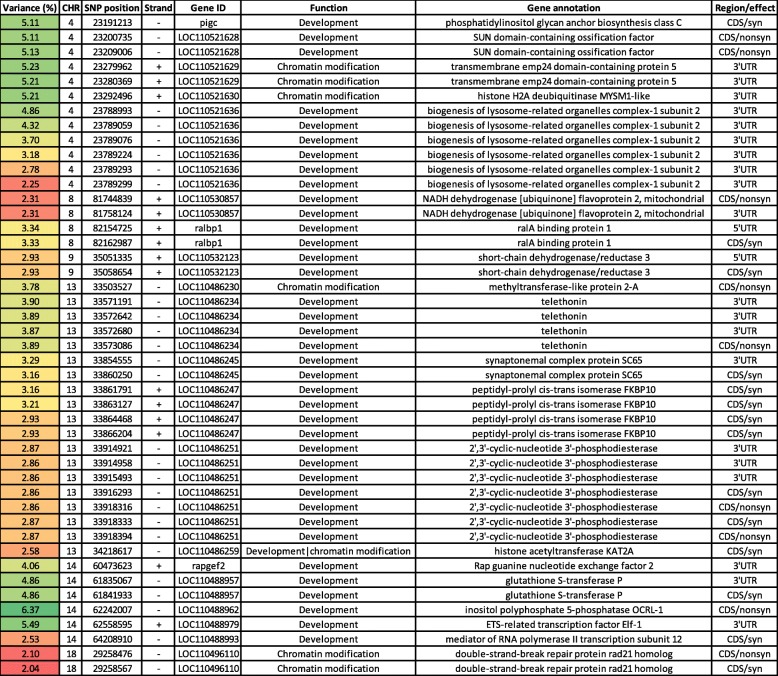
Genomic sliding windows of 50 SNPs explaining at least 2% of the additive genetic variance in bodyweight gain and involved in the development and chromatin modification

A color gradient on the left indicates differences in additive genetic variance explained by windows containing the representative SNP marker (green is the highest and red is the lowest). SNPs are sorted according to their chromosome positions

### Single marker association analysis

**Fig. 3:**
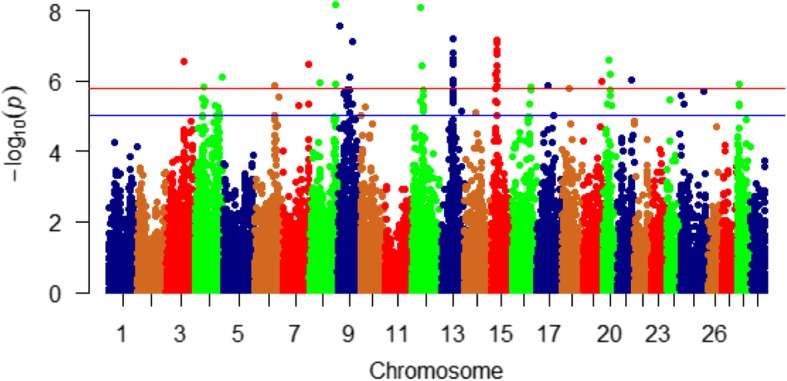
Manhattan plot displaying single SNP markers associated with variations in bodyweight gain using a family-based association analysis (generalized score test). Suggestive and significance threshold *p*-values of 1e-05 and 1.70e-06 are represented by blue and red horizontal lines represent, respectively

Genotyped SNPs were filtered out at a minor allele frequency (MAF) < 0.05 and Hardy–Weinberg equilibrium (HWE) (*p* < 0.001) yielding 29,451 filtered SNPs. In order to identify single SNP markers associated with bodyweight gain, filtered SNPs were subjected to a general linear regression analysis which allows accounting for multiple fixed effects but does not account for familial correlation. Next, residuals of the regression model were regressed on the genetic factors using QFAM, available in PLINK [91], which corrects for the family structure through a special permutation procedure. A total of 738 SNPs were significantly associated with the bodyweight gain (empirical *p*-value < 0.001) following 20,000 permutations. However, a two-stage analysis that calculates residual-outcome from the regression of the outcome on multiple covariates then uses the adjusted-outcome for downstream analysis, showed bias and loss of power in genetic association studies [92, 93]. Therefore, we performed a family-based association analysis using a generalized score test which allows for multiple covariates. A total of 42 SNPs were identified associated with the bodyweight gain after accounting for multiple comparisons (Bonferroni-corrected p “BONF” < 1.70E-06). In order to avoid false positives, the common SNPs between the two-stage and generalized score tests were considered significantly associated with the variation in bodyweight gain (Table S3). In this study, we have identified 33 common SNPs spread over 13 chromosomes with a potential impact on the bodyweight gain (Bonferroni-corrected p “BONF” < 1.70E-06; Table S3 & Fig. 3). One-third of the identified SNPs (33.33%) spanned chromosome 15. SNP-harboring genes were involved in development, cell growth, cell proliferation, and proteolysis. Genes explaining the highest variation in bodyweight gain are coding for thrombospondin-1 (THBS1), microtubule-associated protein 4 (MAP 4), D-3-phosphoglycerate dehydrogenase (PHGDH), calsyntenin-1, nucleolar protein 16 (NOP16), and butyrophilin subfamily 1 member A1 (BTN1A1) (Table 5). THBS1 and MAP 4, ranked at the top of the list, explaining ~ 9 and 6% of the variation in bodyweight gain, respectively. THBS1 is involved in complex biological processes, including angiogenesis and tissue development [94]. Mutation in THBS1 was associated with vascular permeability, accounting for embryonic lethality [75]. Interestingly, seven SNPs spanning ~ 21Kb on chromosome 15, were identified in the gene coding for MAP 4. In mice, blocking the expression of muscle-specific MAP 4 transcript didn’t affect the myoblast growth, but rather severely perturbed the myotube formation indicating a critical role in myogenesis [95]. PHGDH was upregulated in fully differentiated myotubes relative to myoblasts [96]. In addition, three synonymous SNPs were identified in calsyntenin-1, NOP16, and BTN1A1. Each SNP explained ~ 3% of the variation in bodyweight gain. Two intronic SNPs were previously identified in the calsyntenin-1 gene affecting the genetic variance for fillet yield and weight in rainbow trout [22]. NOP16 regulates rRNA production and ribosomal biogenesis. Knockdown of NOP16 dramatically reduced tumor cell growth [97]. BTN1A1 has a function in cell proliferation and development [98].

**Table 5:**
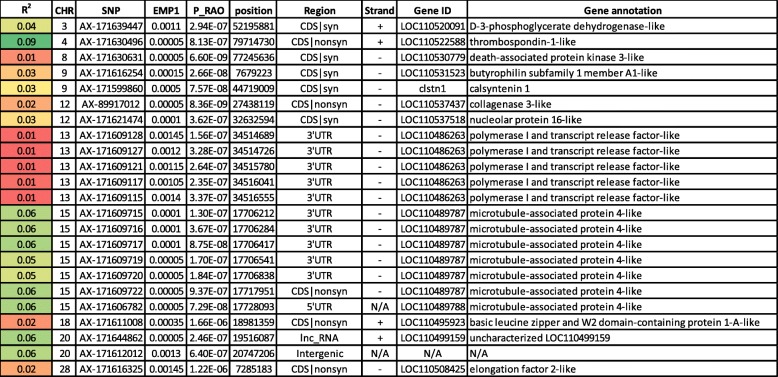
A subset of SNP markers significantly associated with bodyweight gain using two family-based association analyses

A color gradient on the left indicates phenotypic variability explained by each single SNP marker (green is the highest and red is the lowest). SNPs were sorted according to their chromosome positionsNote: EMP1 is pointwise empirical *p*-value estimated using QFAM, whereas P_RAO is the estimated *p*-value using a generalized score test

Three missense mutations were identified in genes coding for collagenase-3 (MMP13), elongation factor 2 (eEF2), and basic leucine zipper and W2 domain-containing protein 1-A (Table 5). Each SNP explained ~ 2% of the variation in bodyweight gain. MMP13 plays a critical role in skeletal system development [99]. eEF2 is a key component in the translation machinery. Inactivation of eEF2 terminates protein synthesis and causes cellular death during mouse embryonic development [100]. An SNP was identified in a gene encoding death-associated protein kinase 3 (Table 5). This protein is involved in the regulation of autophagy [101]. Notably, five 3’UTR mutations were identified in a gene coding for polymerase I and transcript release factor (PTRF/cavin-1) (Table 5). Lack of cavin-1 in mice and humans caused muscular dystrophy [102]. Cavin-1 supports cell proliferation and migration in humans and shows downregulated expression during myogenic differentiation [103]. The remaining SNPs associated with the variation in bodyweight gain are listed in Additional Table (S3).

Single SNP GWA analysis provided an additional set of SNPs, potentially regulating variation in bodyweight gain. In the current study, dividing the genome into chromosomal segments/windows, defined by 50 adjacent markers, outperformed the single-marker analysis in identifying a larger number of SNPs (247 vs. 33 SNPs, respectively) describing the genetic architecture of the studied trait. On chromosome 13, there was a single common significant SNP detected by the two GWA approaches in a gene coding for synaptic vesicle membrane protein VAT-1 homolog (VAT-1). This protein interacts with Talin-1; the key driver of cell migration [104]. Similar results have been previously reported in rainbow trout [25]. Compared to *p*-value based peaks, the highest peaks based on the variance explained depends on allele frequency which means a high-effect SNP of low frequency reduces the variance explained [105]. The WssGBLUB method has been proven to be optimal in livestock populations with a large number of phenotyped animals with a long history of pedigree recording, but lacking genotype data [105]. The two GWA approaches adopted in the current study revealed significant roles of genes related to developmental process in regulating bodyweight gain. Routine use of single-SNP and multi-marker for GWA analysis has been recommended to take advantage of the complete genotype information [106].

Consistent with our data, a previous GWA study in rainbow trout identified small-effect QTL on chromosome 9 that affected additive genetic variance for bodyweight [90]. However, QTL associated with growth rate varied between the studies, and this discrepancy may be due to testing of different populations and gene-by-environment interactions. A 57 K genomic SNP panel has been exploited for GWA analysis, using the same fish population as the current study; the study identified one window on chromosome 5 with small effects on the additive genetic variance for body weight. The window explained 1.38 and 0.95% of the additive genetic variance for body weight at 10 and 13 months, respectively [22]. However, this window was not identified in our study, perhaps, because we considered only windows explaining 2% of the additive genetic variance or more. Several markers, each explaining less than 0.1% of the variance, were identified to be associated with body weight in a GWA study for Atlantic salmon [20]. Fish population, marker density, LD, and size of adjacent SNP windows may, partially, explain the discrepancies in the results obtained from the different studies. In addition, SNPs used in the current study were identified from fish families of extreme phenotypes and thus, perhaps, are more informative for the current GWA analysis [19]. In agreement with previous GWA studies, growth is multifactorial in nature, and growth-related genes regulate development, cell proliferation, energy metabolism, and growth [90, 107]. Overall, the current study further describes the genetic architecture of the studied trait and provides putative markers for breeding candidates that can be used for selection purposes.

## Conclusions

The current GWA study identified growth-related QTL and novel genes associated with the growth rate in rainbow trout. Compared to previous GWA studies in Atlantic salmon and rainbow trout, this work revealed relatively large-effect QTL associated with growth, which appears to be a polygenic trait in nature controlled by many genes on multiple chromosomes. Chromosomes 4 and 14 had the most significant peaks that explained a reasonable proportion of the additive genetic variance for bodyweight gain. The majority of SNP were within genes involved in developmental processes. Intriguingly, the gene harboring the most significant nonsynonymous SNP was previously reported to encode a protein vital to embryonic development. These findings provide a genetic basis that will enhance our understanding of the molecular mechanisms regulating growth in teleost fish as well as provide putative markers that could be prioritized when estimating genomic breeding values for growth rate.

## Methods

### Fish population, tissue sampling, and phenotype

Fish population was previously described [19, 25]. Briefly, fish bodywight data were collected from two consecutive generations (YC 2010 & 2012) produced from the NCCCWA growth-selection breeding program. The NCCCWA breeding program was established in 2002 and has continued for 5 generations of selection producing full-sib families as previously described [10]. Fish used in the current study were harvested from their respective families to allow for measuring other lethal phenotypes, as we previously described [19, 25]. Fish were euthanized with an overdose of MS-222 at a concentration of 300 mg/L. Breeding, hatching, and feeding schedules were previously reported in detail [18].

A total of 789 fish representing 98 families from YC 2010 and 99 families from YC 2012 were phenotyped. For fish sampling of each generation, a single fish from each family was randomly assigned to one of five collection groups (~ 100 fish each) over five consecutive weeks (one group/week). The YC 2010 fish were collected between 410- and 437-days post-hatch with a mean bodyweight of 985 g (SD = 239 g). Fish from the YC 2012 were collected between 446- and 481-days post-hatch with a mean bodyweight of 1803 g (SD = 305 g). The bodyweight gain was calculated as the fish body weight in grams divided by the fish age in days. The pedigree-based heritability *h*^*2*^ (*h*^*2*^*ped*) for growth was estimated according to Zaitlen et al., [108].

### SNP genotyping and quality control

The 50 K transcribed gene SNP-chip used in this study was recently developed and used to identify QTL associated with muscle yield [19], fillet firmness and protein content [25]. Sources of all SNPs used to build the current SNP chip were previously described [18].

As described before, a total of 1728 fish from the NCCCWA growth- and Bacterial Cold Water Disease (BCWD)-selection lines [19] were used to assess the quality of this Affymetrix SNP chip. The SNP chip and sample metrics were reported in our previous publication [19]. Assessment of quality control (QC) and filtration of samples/genotypes have been performed using the Affymetrix SNPolisher software at the default parameters [109]. A total of 789 genotyped fish had available phenotypic data for bodyweight gain and passed the QC; those were used for the current GWA analyses.

### Fifty-SNP window GWA analysis

The Weighted single-step GBLUP (WssGBLUP) has been used to perform GWA analysis, as we previously described [19, 25]. WssGBLUP allows genotyped and ungenotyped animals to be used at the same time, and integrates phenotype, genotype and pedigree information using a mixed model for single-trait analysis as previously described [19, 25]:

$$ y= Xb+{Z}_1a+{Z}_2w+e $$

where y is the vector of the phenotypes, b is the vector of fixed effects including fish data-collection group and hatch-year, **a**, **w**, and **e** are the vectors of direct additive genetic (i.e., animal effect), random family, and residual effects, respectively. The matrices **X**, **Z**_1_, and **Z**_2_ are incidence matrices for the effects contained in **b**, **a**, and **w**, respectively.

This model combines all the relationship information based on pedigree and genotypes into a single matrix (**H**^− 1^):

$$ {H}^{-1}={A}^{-1}+\left[\begin{array}{cc}0& 0\\ {}0& {G}^{-1}-{A}_{22}^{-1}\end{array}\right] $$

where **H**^− 1^ is the inverse of the realized relationship matrix (**H**), **A**^−1^ is the inverse of the relationship matrix based on pedigree information, ${\mathbf{A}}_{22}^{-1}$ is the inverse of the pedigree relationship matrix for genotyped animals only, and **G**^−1^ is the inverse of the genomic relationship matrix.

A modified REMLF90 (AIREMLF90) [110] was used to estimate variances using the Average-Information algorithm. The inbreeding value, accounted for the construction of the inverse of the pedigree relationship matrix, was previously calculated using INBUPGF90 [19, 111]. Pedigree data of 63,808 fish produced from the NCCCWA growth-selection line over five consecutive generations, were fed to INBUPGF90 to calculate the inbreeding value. Using PREGSF90 [111], 35,322 SNPs (70.6%) passed the QC at the following settings; MAF > 0.05, call rate for SNP and samples > 0.90, and HWE < 0.15.

Similar to our previous WssGBLUP analyses [19, 25], two iterations were used in the current analysis where all SNPs were equally weighted (i.e., weight = 1.0) during the first iteration. POSTGSF90 [111] was used to compute SNP effects and weights using sliding windows of 50 adjacent SNPs. The qqman package in R was used to plot the proportion of additive genetic variance explained by every 50 SNPs-genomic window [112].

### Single marker GWA analysis

Two different algorithms were used to perform family-based association analysis of the SNP genotypes with bodyweight gain, and detect signals robust for population stratification. First, QFAM in PLINK version 1.07 [91] was used to perform the family-based association analysis using permutations. QFAM does not allow accounting for the significant contribution of the variables (such as fish data-collection groups and YC) to the predictive power of bodyweight gain model. Therefore, the outcome was adjusted in a linear model in an R package to account for fixed effects (data-collection group and YC) and population stratification using the first two principal components. In the linear model of association using QFAM, the adjusted-outcome was regressed on allele count and the family structure was corrected using 20,000 permutations. Second, a family-based association analysis was performed using a generalized score test [113]. This test accounts for familial correlation using a kinship matrix and allows for multiple covariates. *P*-values were adjusted by Bonferroni correction to account for multiple testing. The qqman package was used to generate a Manhattan plot showing −log_10_ (observed *p*-value) obtained from the GWA analysis.

### Gene annotation and enrichment analysis

SNPs bed file and the rainbow trout genome gff file were provided to Bedtools to annotate the SNPs as previously described [19, 114]. To perform gene enrichment analysis, SNP-harboring genes were uploaded to the Database for Annotation, Visualization, and Integrated Discovery (DAVID) v6.8 [115, 116]. In order to avoid counting duplicated genes, Fisher Exact statistics were calculated based on DAVID gene IDs, which remove redundancies in the original IDs. The list of annotation terms and their associated genes were filtered out based on Fisher Exact < 0.05.

## Supplementary information

**Additional file 1: Table S1.** All QTLs associated with bodyweight gain. **Table S2.** Enriched terms included lysosomal proteins/enzymes and fatty acid biosynthesis (highlighted). **Table S3.** The remaining SNPs associated with the variation in bodyweight gain.

## Data Availability

All datasets generated for this study are included in the manuscript and/or the Additional Files. The genotypes (ped and .map files) and phenotypes are available in our previous publication [25].

## Abbreviations

BCWD: Bacterial Cold Water Disease
DAVID: Database for Annotation, Visualization and Integrated Discovery
DE: Differentially expressed
GO: Gene ontology
GWA: Genome-wide association
HWE: Hardy–Weinberg equilibrium
LD: Linkage disequilibrium
MAF: Minor allele frequency
NCCCWA: USDA National Center of Cool and Cold Water Aquaculture
QC: Quality control
QTL: Quantitative trait loci
SNP: Single nucleotide polymorphism
UTR: Untranslated region
WssGBLUP: Weighted single-step GBLUP
YC: Year class
